# A canine *BCAN* microdeletion associated with episodic falling syndrome

**DOI:** 10.1016/j.nbd.2011.07.014

**Published:** 2012-01

**Authors:** Jennifer L. Gill, Kate L. Tsai, Christa Krey, Rooksana E. Noorai, Jean-François Vanbellinghen, Laurent S. Garosi, G. Diane Shelton, Leigh Anne Clark, Robert J. Harvey

**Affiliations:** a Department of Pharmacology, The School of Pharmacy, 29-39 Brunswick Square, London WC1N 1AX, UK; b Department of Genetics and Biochemistry, College of Agriculture, Forestry, and Life Sciences, 100 Jordan Hall, Clemson University, Clemson, South Carolina 29634-0318, USA; c 55 Bruce Road, Levin 5510, New Zealand; d Biologie Moléculaire, Institut de Pathologie et de Génétique ASBL, 25 Avenue Georges Lemaître, B-6041 Gosselies, Belgium; e Davies Veterinary Specialists, Manor Farm Business Park, Higham Gobion, Hertfordshire, UK; f Department of Pathology, University of California, San Diego, La Jolla, CA 92093-0709, USA

**Keywords:** Brevican, BCAN, Episodic falling syndrome, Cavalier King Charles spaniels, Microdeletion

## Abstract

Episodic falling syndrome (EFS) is a canine paroxysmal hypertonicity disorder found in Cavalier King Charles spaniels. Episodes are triggered by exercise, stress or excitement and characterized by progressive hypertonicity throughout the thoracic and pelvic limbs, resulting in a characteristic 'deer-stalking' position and/or collapse. We used a genome-wide association strategy to map the EFS locus to a 3.48 Mb critical interval on canine chromosome 7. By prioritizing candidate genes on the basis of biological plausibility, we found that a 15.7 kb deletion in *BCAN*, encoding the brain-specific extracellular matrix proteoglycan brevican, is associated with EFS. This represents a compelling causal mutation for EFS, since brevican has an essential role in the formation of perineuronal nets governing synapse stability and nerve conduction velocity. Mapping of the deletion breakpoint enabled the development of Multiplex PCR and Multiplex Ligation-dependent Probe Amplification (MLPA) genotyping tests that can accurately distinguish normal, carrier and affected animals. Wider testing of a larger population of CKCS dogs without a history of EFS from the USA revealed that carriers are extremely common (12.9%). The development of molecular genetic tests for the EFS microdeletion will allow the implementation of directed breeding programs aimed at minimizing the number of animals with EFS and enable confirmatory diagnosis and pharmacotherapy of affected dogs.

## Highlights

► Episodic falling is a canine autosomal recessive paroxysmal hypertonicity disorder. ► We mapped the episodic falling locus to a 3.48 Mb region on canine chromosome 7. ► We found a novel microdeletion in *BCAN* encoding the proteoglycan brevican. ► Multiplex PCR and MLPA tests can distinguish normal, carrier and affected animals. ► *BCAN* mutations may underlie human exercise-induced hypertonicity disorders.

## Introduction

**Fig. 1:**
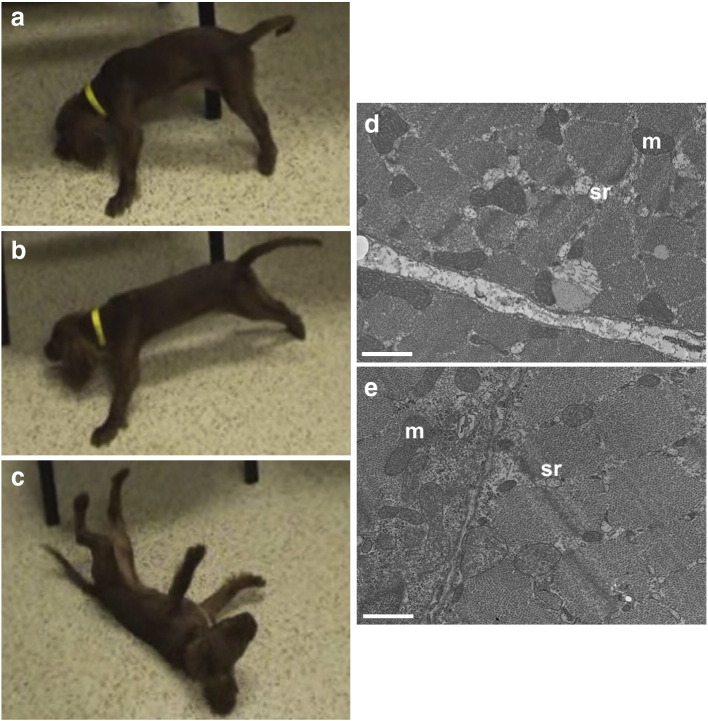
Clinical signs of episodic falling syndrome and muscle pathology. A 5-month-old female Cavalier King Charles spaniel presented with typical episodes of excitement or exercise-induced muscle stiffness (a, hypertonicity) that would involve all four limbs and progress to an usual 'deer-stalking' or 'praying' posture (b), eventually resulting in falling (c). While EFS muscle was normal histologically by light microscopy, electron microscopy (d) revealed that the sarcoplasmic reticulum (sr) appeared dilated and contained finely granular material compared to control muscle (e). Mitochondria (m) and myofibrils were normal in appearance in both tissues. Scale bars = 0.31 μm.

Episodic falling syndrome (EFS) is a well-recognized paroxysmal disorder found in Cavalier King Charles spaniels (CKCS). Episodes begin between fourteen weeks and four years of age and are triggered by exercise, stress, apprehension or excitement (Herrtage and Palmer, 1983). Episodes are of variable frequency and severity but are characterized by progressive hypertonicity involving thoracic and pelvic limbs (Fig. 1a) until the dogs are ultimately immobilized in a characteristic 'deer-stalking' or 'praying' position (Fig. 1b). Stiffening of all four limbs during exercise can cause falling (Fig. 1c), although there is no loss of consciousness or cyanosis. Other clinical signs may include facial muscle stiffness, stumbling, a 'bunny-hopping' gait, arching of the back or vocalization. Curiously, between episodes, dogs appear to be completely normal neurologically. Spontaneous activity was not observed in muscle electrodiagnostic testing, ruling out myotonia congenita (Wright et al*.,* 1986, 1987). Muscle biopsies are typically normal at the light microscopic level, excluding many congenital myopathies. However, EFS has been linked to ultrastructural defects in skeletal muscle including dilatation and proliferation of the sarcoplasmic reticulum, mitochondrial swelling and degeneration (Wright et al., 1986, 1987). EFS has also been compared (Rusbridge, 2005) with startle disease/hyperekplexia, typically characterized by noise- or touch-evoked neonatal hypertonicity due to defects in inhibitory glycine receptor (*GLRA1*, *GLRB*; Shiang et al., 1993; Rees et al., 2002) or glycine transporter GlyT2 (*SLC6A5*) genes (Rees et al., 2006; Harvey et al., 2008). However, a microdeletion in the GlyT2 gene in Irish Wolfhounds results in severe neonatal muscle stiffness and tremor in response to handling (Gill et al., 2011), which is inconsistent with the observed clinical signs of EFS. Comparisons with startle disease may have been made because affected dogs often respond well to the benzodiazepine clonazepam (Garosi et al., 2002), an effective anticonvulsant, anxiolytic and muscle relaxant that is the most effective known treatment for human hyperekplexia (Thomas et al., 2010). However, the carbonic anhydrase inhibitor acetazolamide, used to treat certain types of human episodic ataxia (Tomlinson et al., 2009) and hyperkalemic periodic paralysis (Matthews and Hanna, 2010), also appears to have therapeutic value in the treatment of EFS (http://www.cavalierhealth.org/episodic_falling).

Since a ten-year breeder-led investigation into the inheritance of EFS suggested an autosomal recessive mode of inheritance (http://cavalierepisodicfalling.com/), we used a genome-wide association strategy (Karlsson et al., 2007) to map the EFS locus to a defined region of canine chromosome 7. Candidate gene analysis enabled us to identify a microdeletion affecting the brevican gene (*BCAN*), confirm the deletion breakpoint and develop rapid genotyping tests for EFS.

## Materials and methods

### Light and electron microscopy

For light microscopy, unfixed biopsies from the biceps femoris, vastus lateralis and triceps brachii muscles were collected from five affected CKCS dogs under general anesthesia and frozen in isopentane pre-cooled in liquid nitrogen. Cryosections were cut (8 μm) and the following histochemical stains and reactions performed: hematoxylin and eosin, modified Gomori trichrome, periodic acid Schiff, phosphorylase, esterase, ATPase reactions at pH of 9.8 and 4.3, nicotinamide adenine dinucleotide-tetrazolium reductase, succinic dehydrogenase, acid phosphatase, alkaline phosphatase and oil red O. For electron microscopy, glutaraldehyde-fixed muscle specimens were post-fixed in osmium tetroxide, and dehydrated in serial alcohol solutions and propylene oxide prior to embedding in araldite resin. Thick sections (1 μm) were stained with toludine blue for light microscopy and ultrathin sections (60–90 nm) were stained with uranyl acetate and lead citrate for electron microscopy.

### Study cohort and DNA preparation

Our study cohort comprised: EFS affected—10 animals (6 from the USA, 2 from New Zealand and 2 from the UK); Obligate EFS carriers—8 animals (2 from the USA, 6 from New Zealand); Animals related to carriers or affected dogs—21 animals (7 from the USA, 14 from New Zealand); Controls—CKCS with no EFS history—14 animals (all from the USA). Genomic DNA was isolated from whole blood or buccal cells using the Gentra Puregene Blood Kit (QIAGEN, Valencia, USA). Additional DNA samples from 155 CKCS with no clinical history of EFS and other pure bred-dogs were available from unrelated studies and other sources (e.g. Cornell Medical Genetic Archive: http://www.vet.cornell.edu/research/dnabank/).

### Genome-wide association mapping

Thirteen CKCS genomic DNA samples isolated from blood (five cases, one obligate carrier and seven controls from the USA) were genotyped for 127,000 SNPs on the Affymetrix Canine SNP Array version 2 (http://www.broadinstitute.org/mammals/dog/caninearrayfaq.html). The two main drivers for sample selection were: i) lack of relatedness—i.e., that the animals used for case-control analysis should not share a common ancestor within at least three generations and ii) the quality and quantity of genomic DNA available. Arrays were processed at the Clemson University Genomics Institute (http://www.genome.clemson.edu/) using the GeneChip human mapping 250 K Sty assay kit (Affymetrix, Santa Clara, USA). The GeneChip human mapping 500 K assay protocol was followed, but with a hybridization volume of 125 μl (Karlsson et al., 2007). Raw CEL files were genotyped using Affymetrix Power Tools software. SNPs having > 10% missing data and ≥ 60% heterozygosity were removed. Data for 58,873 SNPs were formatted for PLINK (Purcell et al., 2007) and case/control analyses with 100,000 permutations were performed for five cases and seven controls (the obligate carrier was excluded from analysis).

### PCR and DNA sequencing

PCR primers were designed to amplify exons and flanking splice donor, acceptor and branch-point sites, from gene structures derived *in silico* using the UCSC Genome Browser. For exon-specific primers for *BCAN* and *HAPLN2* exon amplification see Table S1. PCR was performed using 50 ng genomic DNA as template and AccuPrime *Pfx* SuperMix supplemented with betaine for 40 cycles of 94 °C for 1 min, 60 °C for 1 min, 68 °C for 1 min. PCR products were gel purified using a QiaQuick gel extraction kit (QIAGEN, Crawley, UK) for TOPO cloning (pCR4Blunt-TOPO; Invitrogen, Renfrew, UK). Sanger DNA sequencing was performed by DNA Sequencing & Services (MRCPPU, College of Life Sciences, University of Dundee, Scotland) using Applied Biosystems Big-Dye version 3.1 chemistry on an ABI 3730 automated capillary DNA sequencer. DNA sequences were analyzed using Sequencher 4.10 (Gene Codes Corporation, Ann Arbor, USA). For multiplex PCRs, the diagnostic primer set is: EFS1 5′-aaggtcttacacctgcaatgaatag-3′, EFS2 5′-agcaaatgtaaagtcctgtgaccat-3′ and EFS3 5′-agttcacattgtgctctctctactg-3′.

### Multiplex ligation-dependent probe amplification (MLPA) analysis

Five MLPA probe sets were designed corresponding to the promoter region (PR) and exons 1 (5′ UTR), 2, 3 and 4 of the canine brevican gene (*BCAN*; NC_006589 on chromosome 7; Table S2). Criteria for MLPA probe design were as previously described (Schouten et al., 2002). A control probe pair was designed to recognize an unrelated gene (CFTR: NC_006596 on chromosome 14). Probes generated amplification products ranging in size from 88 to 115 bp and had annealing temperatures higher than 70 °C as recommended in RAW Probe software (MRC-Holland, Amsterdam, The Netherlands) using standard MLPA conditions (Schouten et al., 2002). PCR products were analyzed on an ABI 3130XL capillary electrophoresis apparatus (Applied Biosystems, Lennik, Belgium). Normalization of *BCAN*-specific probe signals was performed by dividing the values obtained by the combined signal of the control probes.

## Results

### Light and electron microscopy

Unfixed cryosections of muscle biopsies were histologically normal at the light microscopic level with no abnormalities detected following any of the histochemical stains and enzyme reactions employed. Contrary to previous reports (Wright et al., 1986, 1987) electron microscopy revealed normal myofibrillar and mitochondrial morphology, although swelling of the sarcoplasmic reticulum was confirmed (Figs. 1d,e).

### Genome-wide association mapping and candidate gene resequencing

**Fig. 2:**
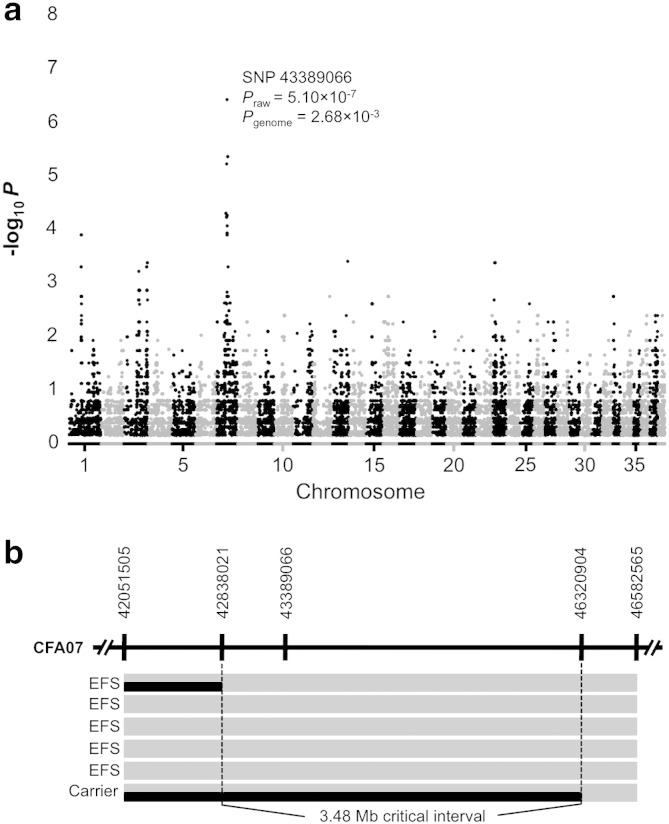
Mapping the episodic falling syndrome locus. (a) –log_10_ of *P*_raw_ values (Y axis) for genome-wide association using five EFS and seven control dogs are plotted for each chromosome (X axis). A single major signal was detected on chromosome 7. (b) A 3.48 Mb critical interval encompassing SNP 43389066 is defined by recombination events in an affected CKCS and an obligate carrier.

**Fig. 3:**
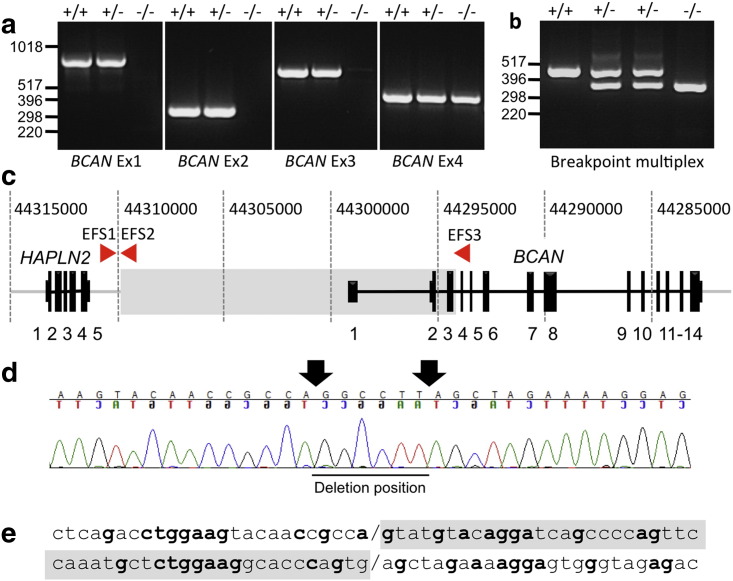
(a) PCR panels for *BCAN* exons 1–4 showing that amplicons for the first three exons of the brevican gene can be generated from genomic DNA from normal (+/+) or obligate carrier (+/−) samples, but cannot be amplified from an equivalent EFS sample (−/−). By contrast, *BCAN* exon 4 can be amplified from all genotypes. (b) Multiplex PCRs with primers flanking the 15.7 kb *BCAN* microdeletion allowed simultaneous detection of the wild-type *BCAN* allele (primers EFS1 + 2, 393 bp) in normal (+/+) or EFS carrier (+/−) animals, while the EFS allele (primers EFS1 + 3, 273 bp) is detected in both EFS carrier and affected (−/−) dogs. Note that the two carriers shown have both wild-type and EFS amplicons, as expected for a heterozygous genotype. (c) Schematic diagram showing the genomic organization of the *HAPLN2* and *BCAN*, the position of the deletion (grey shading) and EFS1-3 primers. (d) Sequence spanning the *BCAN* deletion breakpoint, showing an additional non-homologous inserted sequence indicated by arrows. (e) Alignment of the DNA sequence immediately flanking the deletion breakpoint indicating local microhomology.

A total of 17 single nucleotide polymorphisms (SNPs) were associated with EFS (P_raw_ values ≤ 0.0001) (Table S3). The most significant result was for SNP 43389066 (*P*_raw_ = 5.10 × 10^−7^, *P*_genome_ = 2.68 × 10^−3^) (Fig. 2a). All significant SNPs were located within a 7.2 Mb region on canine chromosome 7. A critical interval of 3.48 Mb (from 7.42838021 to 7.46320904) was delimited by recombinant chromosomes identified in one EFS dog and an obligate carrier (Fig. 2b). In order to identify the mutation associated with EFS, we prioritized several genes for resequencing based on biological plausibility. These encoded ligand-gated or voltage-gated ion channels (*CHRNB2*, *HCN3*, *KCNN3*), mitochondrial (*MRPL24*, *MTSO1*, *MTX1, SLC25A44*), muscle (*MEF2D*, *TPM3*) or brain-expressed proteins (*ARHGEF11*, *BCAN*, *GBA*, *HAPLN2*, *NES*, *RIT1*, *SYT11, UBQLN4*). Curiously, amplification of *BCAN* exons 1, 2 and 3 consistently failed with multiple primer sets when using genomic DNA from affected animals, while DNAs from carriers and unaffected dogs amplified reliably (Fig. 3a). Because no preferential amplification was observed for the adjacent gene, *HAPLN2*—encoding hyaluronan and proteoglycan link protein 2/Bral1 (Hirakawa et al., 2000; Oohashi et al., 2002; Bekku et al., 2010)—we suspected that a microdeletion affecting BCAN regulatory sequences and exons 1–3 was associated with EFS.

### Deletion breakpoint identification and development of diagnostic tests

**Fig. 4:**
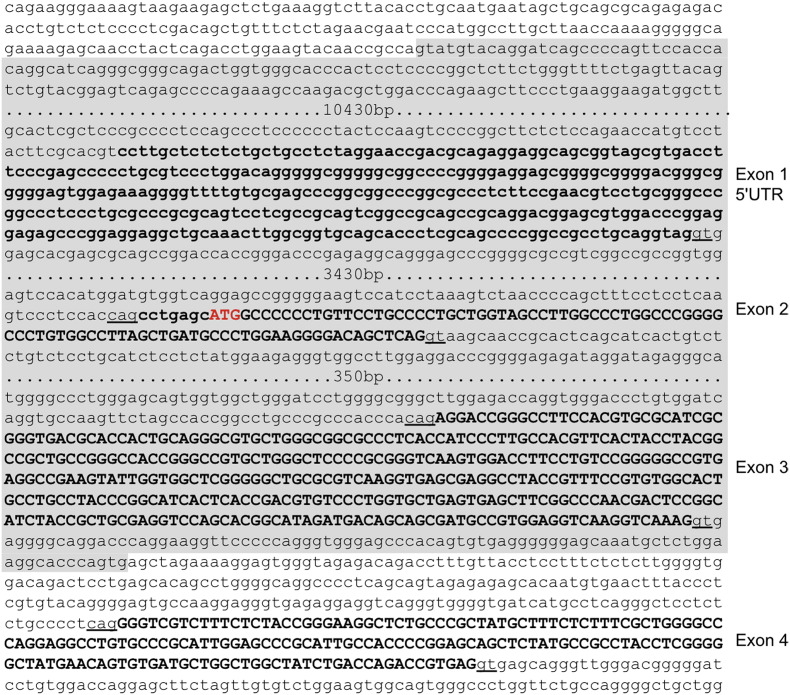
Position of the EFS microdeletion. Genomic DNA from canine chromosome 7 highlighting exons 1–4 of *BCAN* and the position of the 15.7 kb EFS microdeletion (grey shading).

**Fig. 5:**
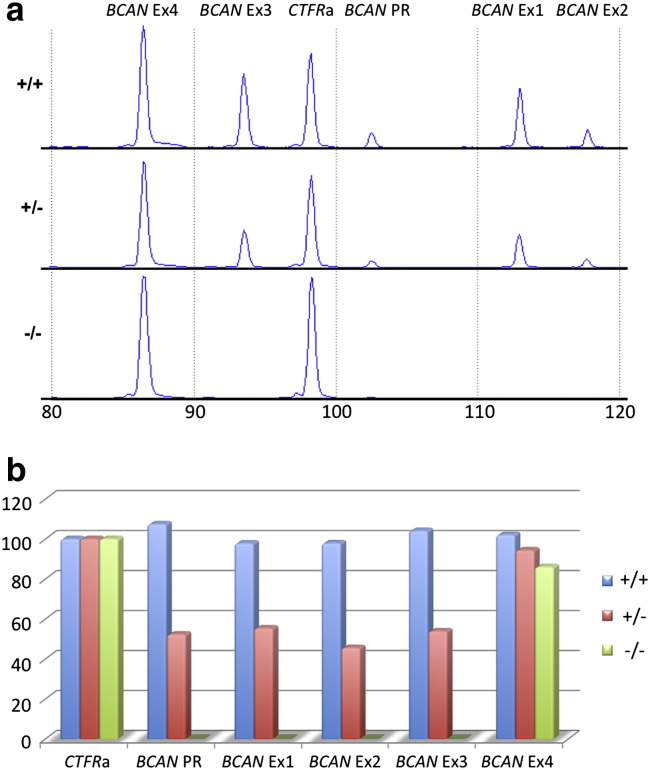
Confirmation of the *BCAN* microdeletion using Multiplex Ligation-dependent Probe Amplification. (a) MLPA analysis revealed robust detection of a control probe (*CFTR*a) and probes for the *BCAN* promoter/regulatory region (PR) and exons 1–4. However, signals for probes PR and exons 1–3 were reduced by 46–55% in heterozygous (+/−) animals and abolished in homozygous animals (−/−), consistent with a loss of probe binding sites in genomic DNA.

Further primer walking experiments enabled us to clone and sequence a DNA fragment containing the deletion breakpoint and develop a multiplex PCR assay that distinguishes between affected, carrier and normal dogs (Fig. 3b). Sequence analysis of the breakpoint amplicon revealed a 15.7 kb microdeletion starting 1.56 kb downstream of *HAPLN2,* encompassing *BCAN* promoter elements and exons 1 (5′ untranslated), 2 and 3, finishing 85 bp downstream of *BCAN* exon 3 (Fig. 3c). The microdeletion amplicon also contained a 6 bp inserted sequence (GGCCTT; Fig. 3d) typical of deletions resulting from non-homologous end joining (NHEJ) or microhomology-mediated end joining (MMEJ). Several regions of microhomology (1–7 bp) were identified in a 30 bp region encompassing the breakpoints (Fig. 3e). Interestingly, 5 of the 6 bp of the reverse complemented inserted sequence align to the largest region of microhomology. We also noted an abundance of short interspersed element (SINE) insertions at the 5' end of the deleted sequence, which could cause the formation of secondary structures that facilitate chromosomal rearrangement (Chuzhanova et al., 2003). We also detected the presence of the BCAN microdeletion (Fig. 4) using MLPA (Schouten et al., 2002) and canine-specific probe sequences (Table S3). This quantitative analysis also confirmed that EFS is associated with a loss of *BCAN* promoter/regulatory elements and exons 1–3 in heterozygous carriers and homozygous affected animals (Fig. 5).

### Rapid genotyping using multiplex genotyping in different dog populations

To assess the prevalence of the EFS microdeletion, we used our multiplex PCR assay to test several CKCS populations and other dog breeds (Table 1). All affected dogs in our study cohort—from the UK (n = 2), USA (n = 6) and New Zealand (n = 2), were homozygous for the *BCAN* microdeletion. In addition, all obligate carriers were heterozygous (n = 2 from the USA and n = 6 from New Zealand). In animals related to affected dogs, we found 9 normal animals and 10 carriers. Interestingly, in this group, two dogs without a classical clinical history of EFS were homozygous for the microdeletion. Lastly, in dogs with no known clinical history of EFS sourced from the USA, the carrier frequency was 12.9% (20/155) suggesting that the EFS microdeletion is present at a high frequency in this population. Notably, the mutation was not detected in multiplex PCRs conducted on control DNA samples from 54 other breeds of dog (Table 1).

## Discussion

The genomic architecture of pure-bred dog lines is ideal for the identification of loci responsible for autosomal recessive traits using genome-wide association mapping (Karlsson et al., 2007; Drögemüller et al., 2009). In this study, we demonstrate that this technique can be used successfully on minimal samples sets, since we located the EFS locus using DNA samples from only five affected and seven breed-matched control dogs. Since a homozygous haplotype spanning 6.35 Mb was identified in affected animals, it is questionable whether further SNP typing would have generated additional useful data. In fact, a single recombination event in an obligate carrier allowed us to narrow the critical interval to 3.48 Mb. This region contained > 100 genes, including ligand-gated ion channels, K^+^ channels, transporters, mitochondrial proteins and several genes known to be involved in neurological disorders in humans. For example, mutations in *CHRNB2* are associated with nocturnal frontal lobe epilepsy (De Fusco et al., 2000) and *TPM3* mutations are associated with nemaline myopathy (Laing et al., 1995). However, many of these genes were rapidly eliminated as candidates due to either: i) poor correlation of EFS clinical signs with the equivalent human disorders or ii) systematic resequencing of the genes. Consistent with the unique clinical signs observed in affected dogs, we discovered that a homozygous microdeletion affecting *BCAN* is associated with EFS in CKCS dogs, confirming that this disorder is inherited in an autosomal recessive manner. This mutation was not detected in control DNA samples from 54 other dog breeds, confirming the unique nature of this genomic rearrangement.

**Fig. 6:**
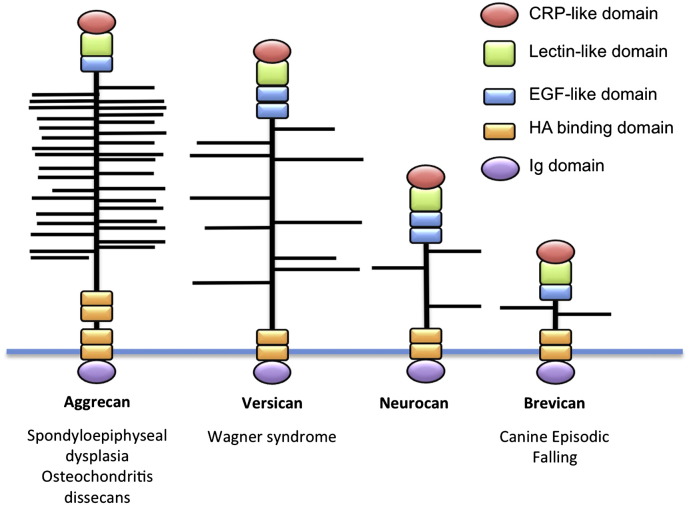
Modular organization of the superfamily of hyaluronan-binding proteins and associated disorders. Mutations in *ACAN*, encoding aggrecan—a major component of cartilage, have been implicated in spondyloepiphyseal dysplasia type Kimberley (Gleghorn et al., 2005) and familial osteochondritis dissecans (Stattin et al., 2010). Mutations in *VCAN*, encoding versican, are associated with Wagner syndrome and erosive vitreoretinopathy, disorders affecting the connective tissue of the eye (Miyamoto et al., 2005). Modified from Maeda et al., 2010.

Brevican belongs to the lectican family of aggregating extracellular matrix (ECM) proteoglycans, which comprises aggrecan, brevican, neurocan and versican. Although mutations in the aggrecan and versican genes (*ACAN*: 15q26.1 and *VCAN*: 5q14.2–14.3) have been linked to different connective tissue disorders (Gleghorn et al., 2005; Stattin et al., 2010; Miyamoto et al., 2005; Fig. 6), no mutations in the brevican or neurocan genes (*BCAN*: 1q23.1 and *NCAN*: 19p13.11) have been identified to date. Brevican and neurocan are highly expressed in the central nervous system, where they are found in specialized extracellular matrix structures called perineuronal nets that play a role in cell adhesion, migration, axon guidance and neuronal plasticity (Brakebusch et al., 2002). Brevican, versican, HAPLN2/BRAL1, tenascin-R and phosphacan are also present at the nodes of Ranvier on large diameter myelinated axons (Bekku et al., 2009, 2010) where cations are accumulated and depleted in the local extracellular nodal region during action potential propagation. The ECM complex at nodes of Ranvier is thought to play a pivotal role in maintaining a local microenvironment, acting as a diffusion barrier for K^+^ and Na^+^ around the perinodal extracellular space (Oohashi et al., 2002; Bekku et al., 2010). Thus, disruption of ECM complexes governing synapse stability and nerve conduction velocity is likely to underlie the EFS phenotype. Certainly, since EFS appears to result from a central nervous system rather than a muscle defect, the associated sarcoplasmic reticulum pathology is likely to be a secondary manifestation of muscle overstimulation (Engel and Banker, 2004).

Interestingly, *BCAN* knockout mice were not reported (Brakebusch et al., 2002) to have a phenotype similar to EFS - although an increased grip-strength was noted, which could be indicative of increased muscle tone. However, it is questionable whether current mouse phenotyping tests will reveal neurological disorders evoked by strenuous exercise, stress, apprehension or excitement, since these conditions are generally avoided in mouse care. It is also noteworthy that EFS clearly shows variable age of onset and penetrance. For instance, we identified two dogs that were homozygous for the *BCAN* microdeletion that had not shown classical clinical signs of EFS in the presence of their owners. Interestingly, similar findings were reported for the dynamin (*DNM1*) mutation (p.R256L) underlying exercise-induced collapse in Labrador retrievers (Patterson et al., 2008). It is plausible that these dogs have not been exposed to sufficient exercise or excitement to trigger an episode, or that genetic or environmental modifying factors (e.g. diet) affect the onset of these disorders. However, it is notable that one of the two asymptomatic dogs in our study was described as 'exercise resistant' by their owner, suggesting that adaptive behavior may occur in dogs that are homozygous for the *BCAN* microdeletion. It must also be emphasized that such non-classical cases are rare—we did not find a single dog that was homozygous for the *BCAN* microdeletion in 155 CKCS with no family history of EFS (Table 1).

In summary, we have shown that an inactivating microdeletion affecting *BCAN* is associated with EFS in CKCS dogs. Identification of the deletion breakpoint has allowed the development of diagnostic tests that have revealed a high prevalence of carriers (12.9%) in clinically unaffected dogs from the USA. These genetic tests (available via Laboklin: http://www.laboklin.co.uk/) will enable future identification of heterozygous animals (which have no discernable phenotypic difference to wild-type animals) allowing directed breeding programs to be implemented, and confirmatory diagnosis and appropriate pharmacotherapy of affected animals. Since this also represents the first report of a genetic disorder involving a neuronal-specific ECM proteoglycan, we suggest that *BCAN* and *NCAN* should be considered as candidate genes for genetic analysis in unresolved cases of human disorders with similar clinical presentations to EFS, such as paroxysmal exercise-induced dyskinesias (Weber and Lerche, 2009) or episodic ataxias (Tomlinson et al., 2009).

## Role of the funding source

The funders had no role in study design, data collection and analysis, decision to publish or preparation of the manuscript. None of the authors declare a conflict of interest.

The following are the supplementary materials related to this article.

Suppl. Table 1 Primer sequences for canine *BCAN and HAPLN2* exon amplification.

Suppl. Table 2 Chromosome 7 single nucleotide polymorphisms associated with EFS.

Suppl. Table 3 Probes for MLPA.

## Figures and Tables

**Table 1:** *BCAN* genotypes in CKCS cohorts and other dog breeds.

Phenotype	Normal	Carrier	Affected
Study CKCS EFS affected	0/10	0/10	10/10
Study CKCS EFS carrier	0/8	8/8	0/8
Study CKCS related to affected or carrier	9/21	10/21	2/21
CKCS with no EFS history	135/155	20/155	0/155
54 dog breeds with no EFS history	93/93	0/93	0/93

Genotypes revealed by multiplex PCRs were determined as described in Materials and methods. Dogs were evaluated on the basis of available clinical data and placed into one of the phenotype categories above. Note that all clinically affected animals were homozygous for the *BCAN* deletion, whilst obligate carriers were heterozygous. As well as wild-type animals and carriers of the *BCAN* deletion, two dogs homozygous for the *BCAN* deletion, which were not reported to have classical clinical signs of EFS, were detected in a cohort of animals related to known EFS dogs. Carriers were also detected in CKCS with no history of EFS, but not in control DNA samples from 54 other dog breeds including: Airedale terrier, Akita Basenji, American Staffordshire Terrier, American Cocker Spaniel, American Eskimo Dog, Australian Shepherd, Akita Basenji, Bernese Mountain Dog, Bluetick Coonhound, Border Collie, Boston Terrier, Boxer, Boykin Spaniel, Briard, Bull Mastiff, Bulldog, Cairn Terrier, Catahoula Leopard Dog, Chihuahua, Collie, Dachshund, Dalmatian, English Setter, English Springer Spaniel, Flat Coated Retriever, German Shepherd, Giant Schnauzer, Golden Retriever, Great Dane, Havanese, Siberian Husky, Irish Setter, Italian Greyhound, Labrador Retriever, Miniature Pinscher, Miniature Poodle, Miniature Schnauzer, New Guinea Singing Dog, Norwegian Elkhound, Petit Basset Griffon Vendeen, Pomeranian, Portuguese Podengo Pequeno, Pug, Pyrenean Shepherd, Schipperke, Shetland Sheepdog, Swedish Vallhund, Tibetan Terrier, Toy Fox Terrier, Weimaraner, Welsh Terrier, West Highland White Terrier, Wire Fox Terrier and Yorkshire Terrier. Where possible, two unrelated dogs were tested for each breed.
