# Supplementary material for: A canine BCAN microdeletion associated with episodic falling syndrome
Source: Neurobiol Dis. 2012 Jan;45(1):130–6. doi: 10.1016/j.nbd.2011.07.014 (PMC3898273; doi:10.1016/j.nbd.2011.07.014)
Supplement: Suppl. Table 1 — Primer sequences for canine BCAN and HAPLN2 exon amplification. [file mmc1.doc]

**Gill et al Suppl. Table 1. Primer sequences for canine *BCAN and HAPLN2* exon amplification.**

| **Gene** | **Exon** | **Forward Primer** | **Reverse primer** |
| --- | --- | --- | --- |
| ***BCAN*** | 1 | cttctctccagaaccatgtcctac | acacagcaagtaagtggcagagtt |
|  | 2 | gagttaacggtgaggttgggacagt | cccacagtgccctctatcctatctc |
|  | 3 | aagatttggggacagatctggagag | ccccaagagagaaaggaggtaacaa |
|  | 4 | ctttctctcttggggtggacagact | ggtcctggatggttgacctgag |
|  | 5 | gggtgtctctgcagaagaaaacaat | gaagacctctggacagcaccg |
|  | 6 | ggcccagagaagccagccta | gggaaacttcagagctcaagtctgt |
|  | 7 | ccacagggaagatgagtgagaattg | ccagcatcactctggacacctt |
|  | 8 | ctcaccctccacagcccctt | ccagagatcatgtgacccagagctt |
|  | 9 | gtgatagctcccaagacaaggagat | gcagggtccaggcttcaggtcta |
|  | 10 | ctctgctggctctctggcat | tgagtgggagagagcaggtga |
|  | 11 | acggacagggaagcagggaa | gcggaggcagaagtgcttgg |
|  | 12 | tgggcctccactcctcatcc | ttacacacatgcggcttgggtc |
|  | 13 | atcccgggtctccaggatca | gagcccagggctactgttggata |
|  | 14 | ccagggctagtgtttggatgagat | ccctcacgtggtcacttcctatg |
| ***HAPLN2*** | 1-2 | gacattccccacacacaccaag | cgcctgtttcgcttcatagtaattg |
|  | 3-5 | tgtctgccggctctccctaa | aggatacagacccatcctgaagcac |
