# Supplementary material for: A canine BCAN microdeletion associated with episodic falling syndrome
Source: Neurobiol Dis. 2012 Jan;45(1):130–6. doi: 10.1016/j.nbd.2011.07.014 (PMC3898273; doi:10.1016/j.nbd.2011.07.014)
Supplement: Suppl. Table 2 — Chromosome 7 single nucleotide polymorphisms associated with EFS. [file mmc2.doc]

**Gill et al Suppl. Table 3. Probes for MLPA**

| **Probe** | **Sequence** | **Tm°C** | **%GC** | **Size** |
| --- | --- | --- | --- | --- |
| **CFTRa** | GGGTTCCCTAAGGGTTGGAGCTCCATTGCAATCTACCTAGCCATTG | 72 | 48 | 100 |
|  | P-GCTTATGCCTTCTCTTTATCATGAGGCCGCTTCTAGATTGGATCTTGCTGGCAC | 75 | 48 |  |
| ***BCAN* PR** | GGGTTCCCTAAGGGTTGGAGGATTGGGTGCCCTGTTTGG | 72 | 60 | 104 |
|  | P-CTCGCCACAGGGAGCCTTCTGCGCATTTCCAAGTGGTCACTGTCTAGATTGGATCTTGCTGGCAC | 72 | 65 |  |
| ***BCAN***  **Exon 1** | GGGTTCCCTAAGGGTTGGACTGGTCTAGCCTCTAGGAACCGACGCAGAG | 70 | 62 | 111 |
|  | P-GAGGCAGCGGTAGCGTGACAGGCTGGGGAAGAGCAAAAATCACGGAGTCTCTAGATTGGATCTTGCTGGCAC | 71 | 68 |  |
| ***BCAN***  **Exon 2** | GGGTTCCCTAAGGGTTGGACTATGCAAGGTGTGGCCTTAGCTGATGCCCTG | 72 | 59 | 115 |
|  | P-GAAGGGGACAGCTCAGGTAAGCAGGAGCCCGAGGGGTGTCCTCTAGATTGGATCTTGCTGGCAC | 70 | 59 |  |
| ***BCAN***  **Exon 3** | GGGTTCCCTAAGGGTTGGAGCCATCTACCGCTGCGAGGTCCAGCACG | 77 | 64 | 96 |
|  | P-GCATAGATGACAGCAGCGATGCCGTATCTAGATTGGATCTTGCTGGCAC | 72 | 57 |  |
| ***BCAN***  **Exon 4** | GGGTTCCCTAAGGGTTGGAGGGGCTATGAACAGTGTGATGCTG | 72 | 54 | 88 |
|  | P-GCTGGCTATCTGACCAGACCGTTCTAGATTGGATCTTGCTGGCAC | 71 | 59 |  |

Key: P- = 5' phosphate group, Blue: 5' PCR primer target, Green: 3' PCR primer target, Red = stuffer

Observed migration: *BCAN* Ex3 86 bp; *BCAN* Ex2 94 bp; *CFTR*a control 98 bp; *BCAN* PR 102 bp; *BCAN* Ex0 113 bp; *BCAN* Ex1 118 bp; *BCAN* Ex0 113 bp.
