# Supplementary material for: A canine BCAN microdeletion associated with episodic falling syndrome
Source: Neurobiol Dis. 2012 Jan;45(1):130–6. doi: 10.1016/j.nbd.2011.07.014 (PMC3898273; doi:10.1016/j.nbd.2011.07.014)
Supplement: Suppl. Table 3 — Probes for MLPA. [file mmc3.doc]

**Gill et al Suppl. Table 2. Chromosome 7 single nucleotide polymorphisms associated with EFS.** A total of 17 SNPs on canine chromosome 7 were associated with EFS (*P*_raw_ values ≤ 0.0001).

| **SNP** | **P-value** |
| --- | --- |
| 7.43389066 | 5.10×10^-7^ |
| 7.46204875 | 5.61×10^-6^ |
| 7.46283892 | 5.61×10^-6^ |
| 7.42051505 | 7.65×10^-6^ |
| 7.39115202 | 6.12×10^-5^ |
| 7.39142101 | 6.12×10^-5^ |
| 7.43140878 | 6.73×10^-5^ |
| 7.43197311 | 6.73×10^-5^ |
| 7.43230030 | 6.73×10^-5^ |
| 7.43298329 | 6.73×10^-5^ |
| 7.43302986 | 6.73×10^-5^ |
| 7.43867010 | 6.73×10^-5^ |
| 7.43917092 | 6.73×10^-5^ |
| 7.44014865 | 6.73×10^-5^ |
| 7.44059025 | 6.73×10^-5^ |
| 7.41416845 | 7.19×10^-5^ |
| 7.42027729 | 7.19×10^-5^ |
